# Nitrogen fixation estimates for the Baltic Sea indicate high rates for the previously overlooked Bothnian Sea

**DOI:** 10.1007/s13280-020-01331-x

**Published:** 2020-04-20

**Authors:** Malin Olofsson, Isabell Klawonn, Bengt Karlson

**Affiliations:** 1 Research and Development, Oceanography, Swedish Meteorological and Hydrological Institute, Sven Källfelts Gata 15, 426 71 Västra Frölunda, Gothenburg, Sweden; 2 Department of Experimental Limnology, IGB-Leibniz-Institute of Freshwater Ecology and Inland Fisheries, Alte Fischerhütte 2, OT Neuglobsow, Stechlin, 16775 Berlin, Germany; 3 Present Address: Department of Aquatic Sciences and Assessment, Swedish University of Agricultural Sciences, Uppsala, Sweden

**Keywords:** *Aphanizomenon*, Baltic Sea, *Dolichospermum*, Filamentous cyanobacteria, Nitrogen fixation, *Nodularia spumigena*

## Abstract

Dense blooms of diazotrophic filamentous cyanobacteria are formed every summer in the Baltic Sea. We estimated their contribution to nitrogen fixation by combining two decades of cyanobacterial biovolume monitoring data with recently measured genera-specific nitrogen fixation rates. In the Bothnian Sea, estimated nitrogen fixation rates were 80 kt N year^−1^, which has doubled during recent decades and now exceeds external loading from rivers and atmospheric deposition of 69 kt year^−1^. The estimated contribution to the Baltic Proper was 399 kt N year^−1^, which agrees well with previous estimates using other approaches and is greater than the external input of 374 kt N year^−1^. Our approach can potentially be applied to continuously estimate nitrogen loads via nitrogen fixation. Those estimates are crucial for ecosystem adaptive management since internal nitrogen loading may counteract the positive effects of decreased external nutrient loading.

## Introduction

Anthropogenic pressures and climate change are increasingly affecting Earth’s ecosystems in a synergistic manner (Steffen et al. 2015). Ongoing climate change in combination with external nutrient loads (especially phosphorus) from human activities will enhance the growth of nitrogen-fixing cyanobacteria in aquatic environments (Paerl and Huisman 2008; Wannicke et al. 2018). Nitrogen fixation allows diazotrophic organisms to bypass nitrogen limitation and their contributions to global nitrogen cycling is continuously a research focus (Benavides et al. 2018; Landolfi et al. 2018; Tang et al. 2019; Wang et al. 2019). In addition, up to half of newly fixed nitrogen is released as bioavailable reactive nitrogen compounds (e.g., ammonium and dissolved organic nitrogen) (Wannicke et al. 2009; Ploug et al. 2010, 2011; Loick-Wilde et al. 2019), which are quickly assimilated by co-occurring organisms (Adam et al. 2016; Klawonn et al. 2019). Hence, the presence, activity, and future distribution of nitrogen-fixing cyanobacteria pose an impact to whole ecosystem function (e.g., Karlson et al. 2015).

The Baltic Sea, one of the largest semi-enclosed water bodies in the world, has an early history of multi-stressors (Reusch et al. 2018). The region also has a long history of monitoring programs, several initiated by the Baltic Marine Environment Protection commission HELCOM. Here, dense blooms of diazotrophic filamentous cyanobacteria are formed every summer in the near-surface water mass (Larsson et al. 2001; Wasmund et al. 2001). These blooms have increased along with elevated external nutrient loads during the recent century (Finni et al. 2001). Despite successful reductions in external loads during the recent decades (Gustafsson et al. 2012), the abundance of filamentous cyanobacteria is still increasing (Kahru and Elmgren 2014). To further reduce the external nutrient loads to the Baltic Sea, neighboring countries have agreed upon the Baltic Sea Action Plan (HELCOM 2007) where HELCOM also provide regular reports of estimated current nutrient loads and suggested allowable limits (HELCOM 2018).

Pelagic nitrogen fixation in the Baltic Sea is dominated by three genera of filamentous cyanobacteria (Klawonn et al. 2016). The toxic *Nodularia spumigena* are mostly common in the southern parts and the non-toxic *Aphanizomenon* sp. and *Dolichospermum* spp. dominate in the northern parts (Wasmund et al. 2017). Two decades ago, nitrogen fixation within the Baltic Proper (southern parts of the Baltic Sea) was estimated to equal the input from riverine runoff and be double the atmospheric load (Larsson et al. 2001; Wasmund et al. 2001), while estimates for the northern regions of the Baltic Sea are still lacking. These parts are often overlooked, partly since *Aphanizomenon* sp. is found in sub-surface waters making it challenging to detect on satellite images (Hajdu et al. 2007). Despite low attention, monitoring studies indicate that the abundance of filamentous cyanobacteria has increased also in the Baltic Sea northern regions during recent decades (Jaanus et al. 2011; Kahru and Elmgren 2014; Andersson et al. 2015; Olofsson et al. 2020).

The aims of this study were to (A) demonstrate the spatial and temporal distribution of diazotrophic filamentous cyanobacteria at a genus level across the Baltic Sea, reaching from the southern to the northern parts, during the two recent decades (1999–2017), (B) estimate basin-wide annual internal nitrogen load via nitrogen fixation, and (C) provide an alternative approach to previous studies (e.g., nitrogen budget calculations from total nitrogen, conceptual models, and upscaling of nitrogen fixation measurements) for estimates of internal nitrogen loads, using monitoring data combined with empirical genera-specific nitrogen fixation rates.

## Materials and methods

### Study area and monitoring data

**Fig. 1:**
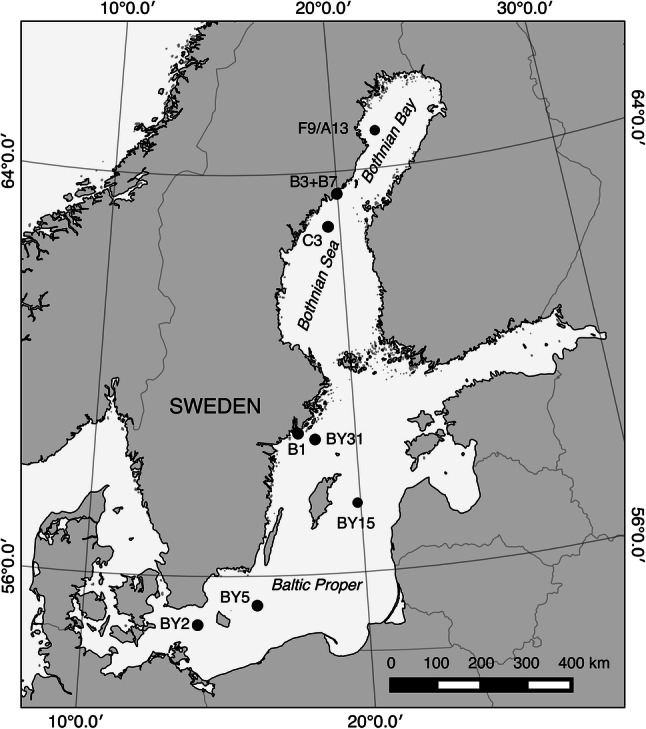
Map of the Baltic Sea and the Swedish National monitoring stations in the Baltic Proper (Arkona BY2, Bornholm BY5, Gotland deep BY15, Askö B1, and Landsort deep BY31), Bothnian Sea (Offshore C3 and Öre Estuary B3 + B7), and Bothnian Bay (Offshore F9/A13)

The Baltic Sea is a brackish semi-enclosed body comprising several sub-basins, each with distinct salinity and nutrient loads. The Swedish National Marine Monitoring Program includes regular sampling of phytoplankton as well as physical and chemical parameters. These data are made available by the Swedish National Oceanographic Data Centre at the Swedish Hydrological and Meteorological Institute via open access at https://sharkweb.smhi.se. The present study includes data from eight monitoring stations from the southern to the northern basins (Fig. 1) compiled for the years 1999–2017. This period of time was selected for its data coverage of all eight monitoring stations.

The monitoring data includes phytoplankton biovolume, which is calculated based on cell numbers and filament sizes (Olenina et al. 2006). Biovolume of diazotrophic filamentous cyanobacteria were compiled for the two decades to determine the spatial and temporal distribution of the three taxa *Nodularia spumigena*, *Aphanizomenon* sp., and *Dolichospermum* spp. The biovolumes (mm^3^ L^−1^) from stations B1 and BY31 were multiplied by 2 to adjust for the tube-sampling depths of 0–20 m as compared to the other stations of 0–10 m. This is justified since filamentous cyanobacteria are mainly found at or above ca. 10 m (Hajdu et al. 2007; Karlson, pers. comm.). The biovolume of each cyanobacterial genus was calculated as monthly averages across all years for each station to demonstrate the seasonal dynamics, and since most of the stations are sampled more frequently during summer (up to 2–3 times per month), the number of n will sometimes be larger than number of years and stations multiplied (Table 1). The monthly means during June–August were also used to provide annual variability in biovolumes of the three genera of during summer for the Baltic Proper (five stations) and Bothnian Sea (two stations). Cyanobacterial biovolumes were aggregated in Plankton Toolbox (version 1.3.1, Karlson et al. 2016).

**Table 1:** Monitoring stations and number of samples per month during the period (1999–2017) for biovolumes of filamentous cyanobacteria

Month	F9/A13	B3 + B7	C3	BY31	B1	BY15	BY5	BY2
January	15	16	16	15	18	16	14	17
February	9	22	16	15	19	14	14	15
March	10	33	7	47	42	22	20	20
April	15	31	17	71	77	20	19	20
May	22	43	21	40	40	16	16	16
June	24	39	23	40	39	17	20	19
July	18	17	21	46	46	24	22	23
August	20	31	18	45	46	23	23	23
September	16	27	12	38	38	16	16	16
October	11	31	10	21	36	16	18	17
November	8	16	9	23	20	17	19	18
December	18	17	15	14	19	12	13	12

In addition, carbon concentrations (derived from biovolume conversion according to Menden-Deuer and Lessard 2000) were extracted from the database for the three cyanobacteria. These data were used to compare the relative biomass between nitrogen-fixing cyanobacteria and other autotrophic organisms (multiplied by 2 at B1 and BY31) across seasons.

### Estimating nitrogen fixation

Nitrogen fixation rates were estimated using empirical in situ genera-specific measurements of nitrogen fixation rates and genera-specific cell sizes from Klawonn et al. (2016). The measurements were performed monthly during summer (June–August) in 2012 and 2013 at the coastal station (B1) and offshore station (BY31), respectively. These measurements were performed using stable isotope tracer incubations, with ^15^N_2_ gas pre-dissolved in seawater (Montoya et al. 1996; Klawonn et al. 2015), in combination with secondary-ion mass spectrometry (SIMS) (Musat et al. 2012). Genera-specific nitrogen fixation rates were obtained from the SIMS measurements where individual cells of filamentous cyanobacteria are analysed (number of cells analyzed per genus is provided in Table 2). For each cell, the measurements reveal the stable isotope ratio of ^15^N_2_ gas transformed into organic matter. This cell-specific nitrogen fixation rates for each genus were thereafter divided by mean cell size of each taxa (from Klawonn et al. 2016), to a genera-specific fixation rate per biovolume of cell (µmol N mm^−3^). The genera-specific rates were thereafter averaged over seasons (June–August), stations (B1 and BY31), and depths (0–7 or 0–12 m). The according mean rates ranged from 0.66 to 1.2  µmol mm^−3^ day^−1^ (Table 2), and were used to estimate nitrogen fixation rates (mmol N m^−3^ day^−1^) as average for the upper 10 m:

**Table 2:** Genera-specific nitrogen fixation per volume (µmol mm^−3^ day^−1^) and number of cells analyzed using SIMS for the filamentous cyanobacteria *Aphanizomenon* sp., *Nodularia spumigena,* and *Dolichospermum* spp. (from Klawonn et al. 2016) used to estimate nitrogen fixation rates based on monitoring data. Rates are given as mean ± standard error

Taxa	Nitrogen fixation (µmol mm^−3^ day^−1^)	Number of cells analyzed
*Aphanizomenon* sp.	0.66 ± 0.11	1227
*Nodularia spumigena*	0.68 ± 0.14	1129
*Dolichospermum* spp.	1.20 ± 0.25	1640

1 $${\text{Estimated}}\, {N}_{2}\, {\text{fixation}}={\text{Genera}}-specific\,{ N}_{2}\, {\text{fixation}} \times {\text{Biovolume}}_{({\text{sample}})}$$

where genera-specific nitrogen fixation is the average nitrogen fixation rate per taxa normalized to the average genera-specific cell volume (µmol mm^−3^ day^−1^; Table 2). The genera-specific fixation was multiplied with the genera-specific biovolume (mm^3^ L^−1^) of cyanobacteria obtained from monitoring data. Using biovolume instead of cell numbers, we normalize for large variations in cell sizes. For seasonal dynamics, the nitrogen fixation rates were depth-integrated over 0–10 m to estimate areal nitrogen fixation rates at each station as a monthly mean across the years 1999–2017 (mmol N m^−2^ day^−1^). For the annual estimates, monthly mean values of nitrogen fixation per day were calculated across stations within each basin (five stations for the Baltic Proper and two for the Bothnian Sea, see Fig. 1). This monthly mean value for each basin was multiplied by 30 to attain a total estimate for each given month. The monthly estimates were thereafter summarized to an annual estimate per basin (mmol N m^−2^ year^−1^) and nitrogen load for the Baltic Proper (211 000 km^2^) and the Bothnian Sea (79 000 km^2^) by multiplying with surface area. Due to low frequency of sampling in the Bothnian Sea, the monthly mean values were calculated across six or seven years (sampling period divided by three). The estimated internal nitrogen inputs (via nitrogen fixation) were compared with the total external input of nitrogen (rivers, direct point-sources [e.g., from waste water or industry], and atmospheric deposition) provided by HELCOM (2018). All data were processed and plotted using the package ´Tidyverse` in R (R Core Team 2016; Wickham 2017).

## Results

### Spatial and temporal distribution of cyanobacteria

**Fig. 2:**
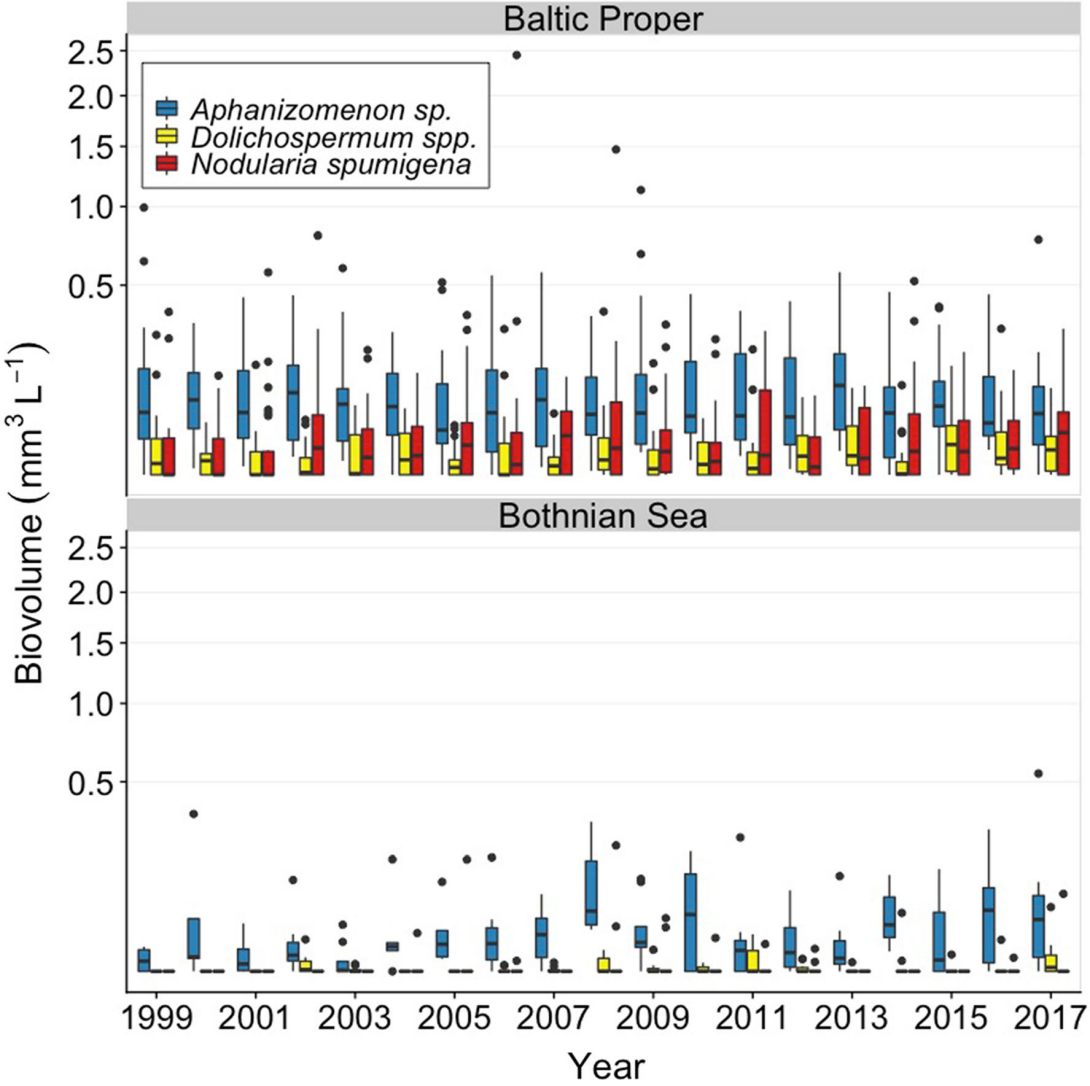
Biovolume (mm^3^ L^−1^) during summer (June–August) of the three genera of filamentous cyanobacteria for 1999–2017 in the Baltic Proper and the Bothnian Sea, across five and two stations, respectively. Box-whisker plots include median values, the 25th and 75th percentile, respectively, and the outer values represent either 1.5 or 3 times beyond the end of the box. Please note that the *y*-axes are square root transformed. Data derived from the Swedish National Oceanographic Data Centre

**Fig. 3:**
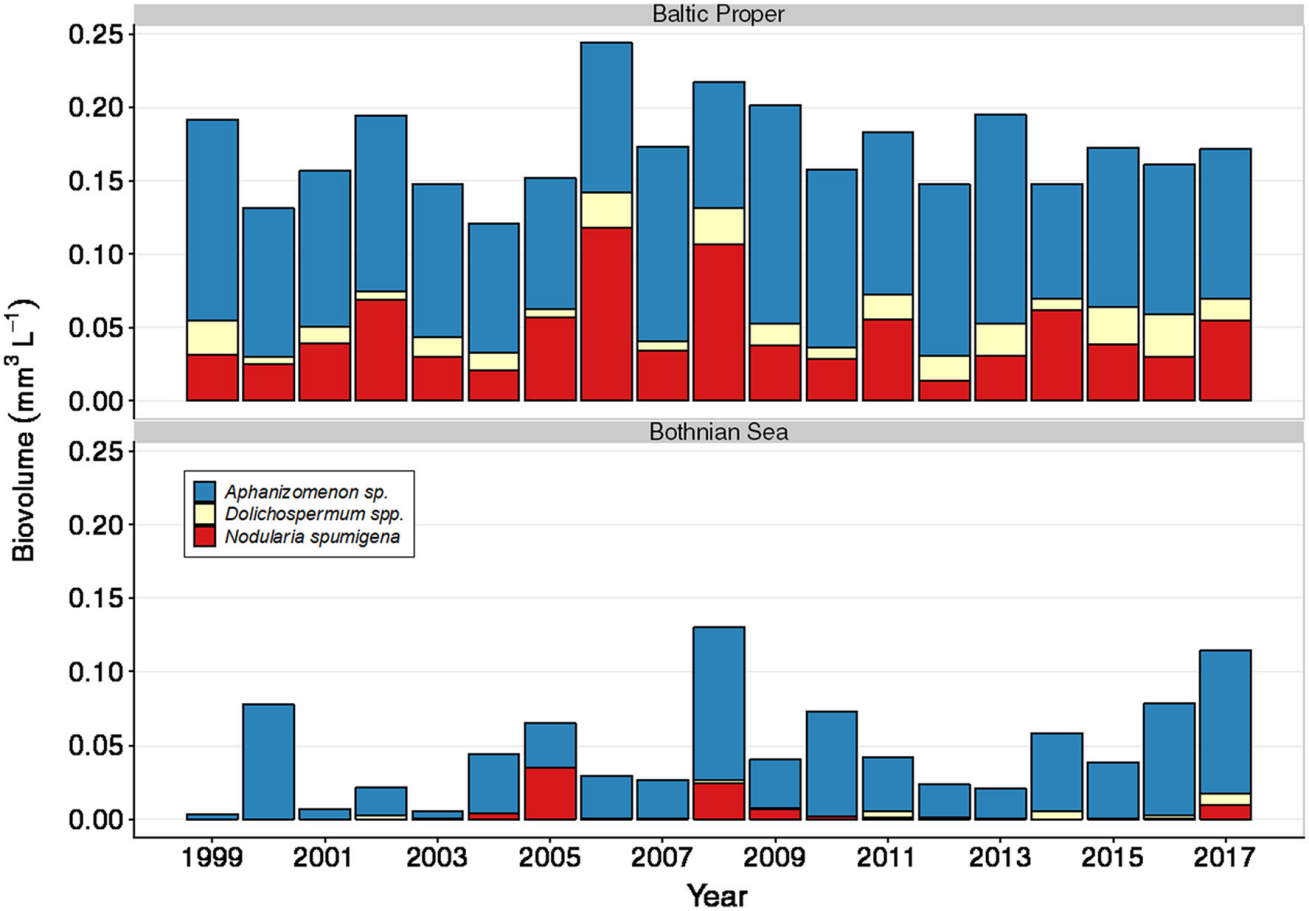
Total biovolume (mm^3^ L^−1^) as average during summer (June–August) of the three genera of filamentous cyanobacteria for 1999–2017 for the Baltic Proper (five stations) and the Bothnian Sea (two stations). Data derived from the Swedish National Oceanographic Data Centre

**Fig. 4:**
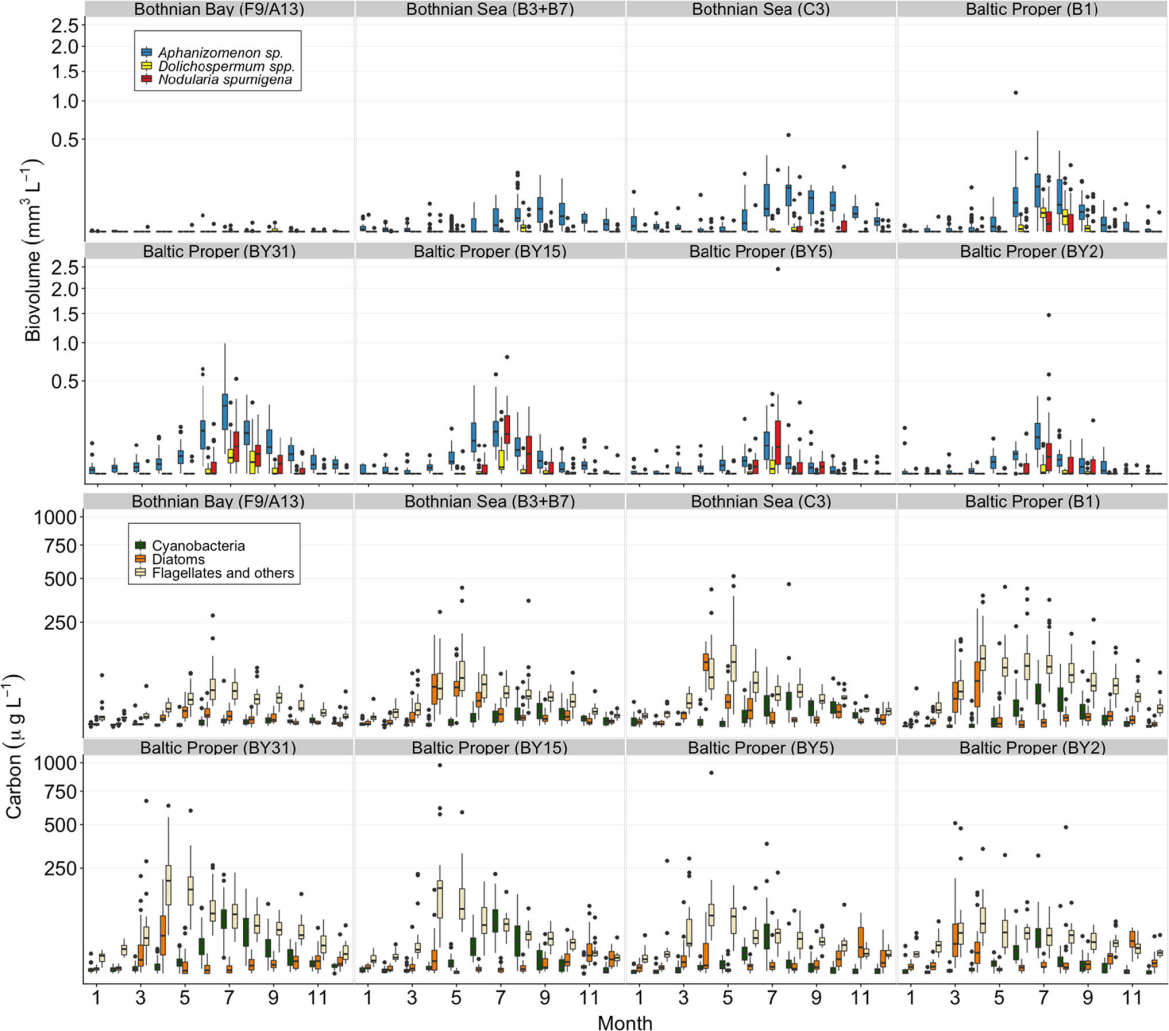
Upper set of panels includes biovolume (mm^3^ L^−1^) of the three genera of filamentous cyanobacteria and the lower set of panels includes carbon concentration (µg L^−1^) for the groups of cyanobacteria (filamentous), diatoms, and flagellates and others (autotrophic flagellates and other autotrophic organisms), for each month at the different stations across the two recent decades. Box-whisker plots include median values, the 25 th and 75 th percentile, respectively, and the outer values represent either 1.5 or 3 times beyond the end of the box. Please note that the *y*-axes are square root transformed. Data derived from the Swedish National Oceanographic Data Centre

*Aphanizomenon* sp. was the cyanobacteria genus with the highest biovolume in the Northern Baltic Proper and Bothnian Sea. Both *Aphanizomenon* sp. and *Nodularia spumigena* were common in the in the Southern Baltic Proper, and *Dolichospermum* spp. was overall less abundant as compared to the other two genera (Figs. 2, 3, 4). The highest biovolumes of *Aphanizomenon* sp. were found at station B1 and BY31 in the Northern Baltic Proper. In the Bothnian Sea, high biovolumes of filamentous cyanobacteria were also found at station C3, while they were almost non-detectable in the further northern Bothnian Bay (summer mean < 0.001 mm^3^ L^−1^). The seasonal pattern was similar across stations. In the winter, only *Aphanizomenon* sp. was present, though at low abundance. Then, *Aphanizomenon* sp. increased in abundance earlier in the spring than *N. spumigena* (Fig. 4). The annual average biovolume of filamentous cyanobacteria for all stations within the Baltic Proper and Bothnian Sea during summer showed a large variation both within and between years. In the Bothnian Sea, mostly dominated by *Aphanizomenon* sp., the cyanobacteria biovolume occurred at higher abundances during recent years as compared to the initial years of the observed period (Figs. 2 and 3).

The carbon biomass of cyanobacteria varied between 12 and 57% out of the total autotrophic community (> 3 μm) during summer (June–August) in the Baltic Proper (Fig. 4). Here, the greatest values of absolute cyanobacterial biomass and the cyanobacterial biomass as a fraction of total biomass were observed in July at stations BY15 and BY2. In the Bothnian Sea, this occurred at station C3 in August. Overall, the carbon biomass of cyanobacteria accounted for 8–58% of total autotrophic biomass (> 3 µm) in this region. In the Bothnian Bay, the biomass proportion of cyanobacteria never exceeded 10% during summer, where the overall biomass of autotrophic organisms > 3 µm was much lower than at other stations, and dominated by autotrophic flagellates. The peak in diatom biomass during spring appeared about one month later in the Bothnian Bay and the Bothnian Sea (April) as compared to the Baltic Proper (March). Diatoms were generally low in abundance at stations BY15 and BY5, compared to other autotrophic organisms (Fig. 4).

### Estimated nitrogen fixation

**Fig. 5:**
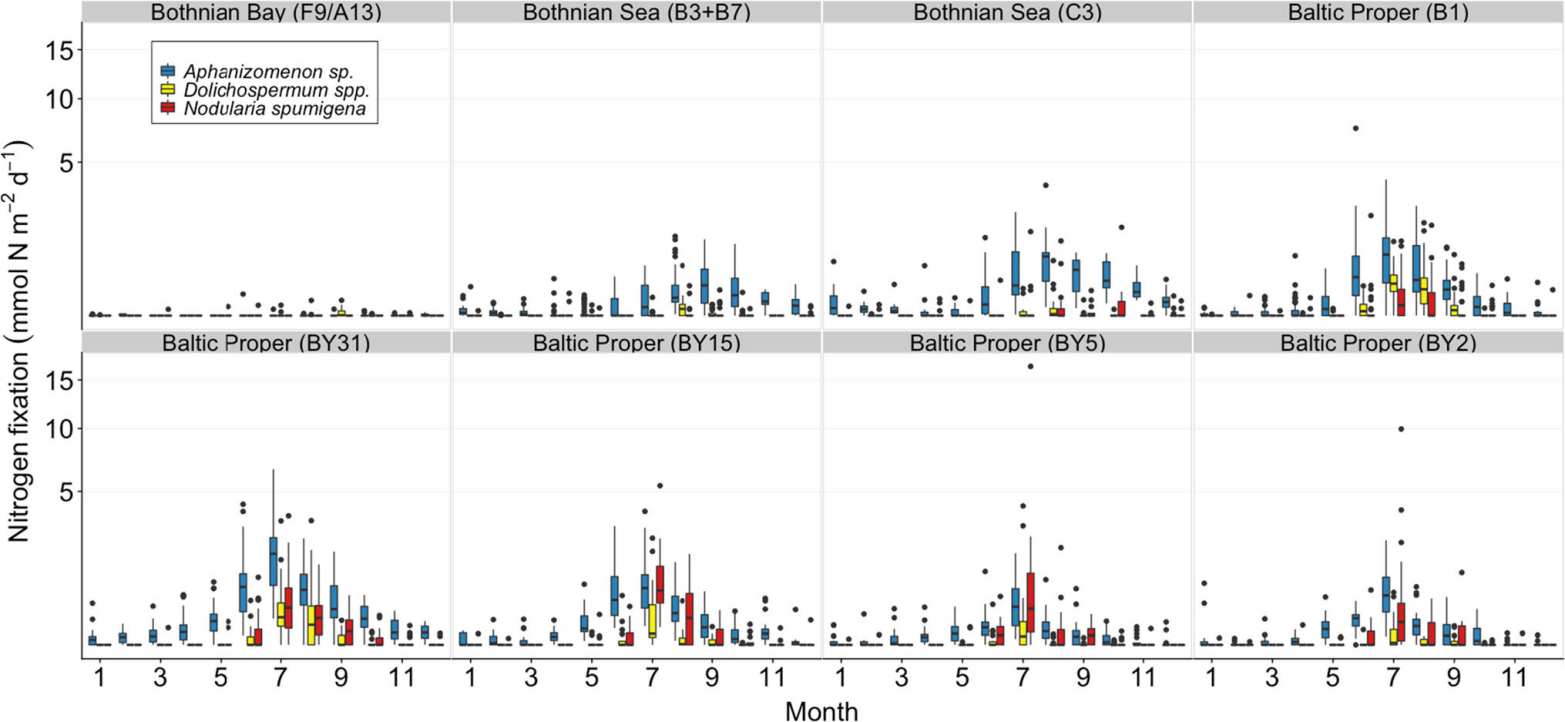
Estimated nitrogen fixation rates (mmol N m^−2^ day^−1^) per month during the period 1999–2017 for all stations and the three filamentous cyanobacteria taxa *Aphanizomenon* sp., *Dolichospermum* spp, and *Nodularia spumigena*. Box-whisker plots include median values, the 25 th and 75 th percentile, respectively, and the outer values represent either 1.5 or 3 times beyond the end of the box. Please note that the *y*-axes are square root transformed

**Fig. 6:**
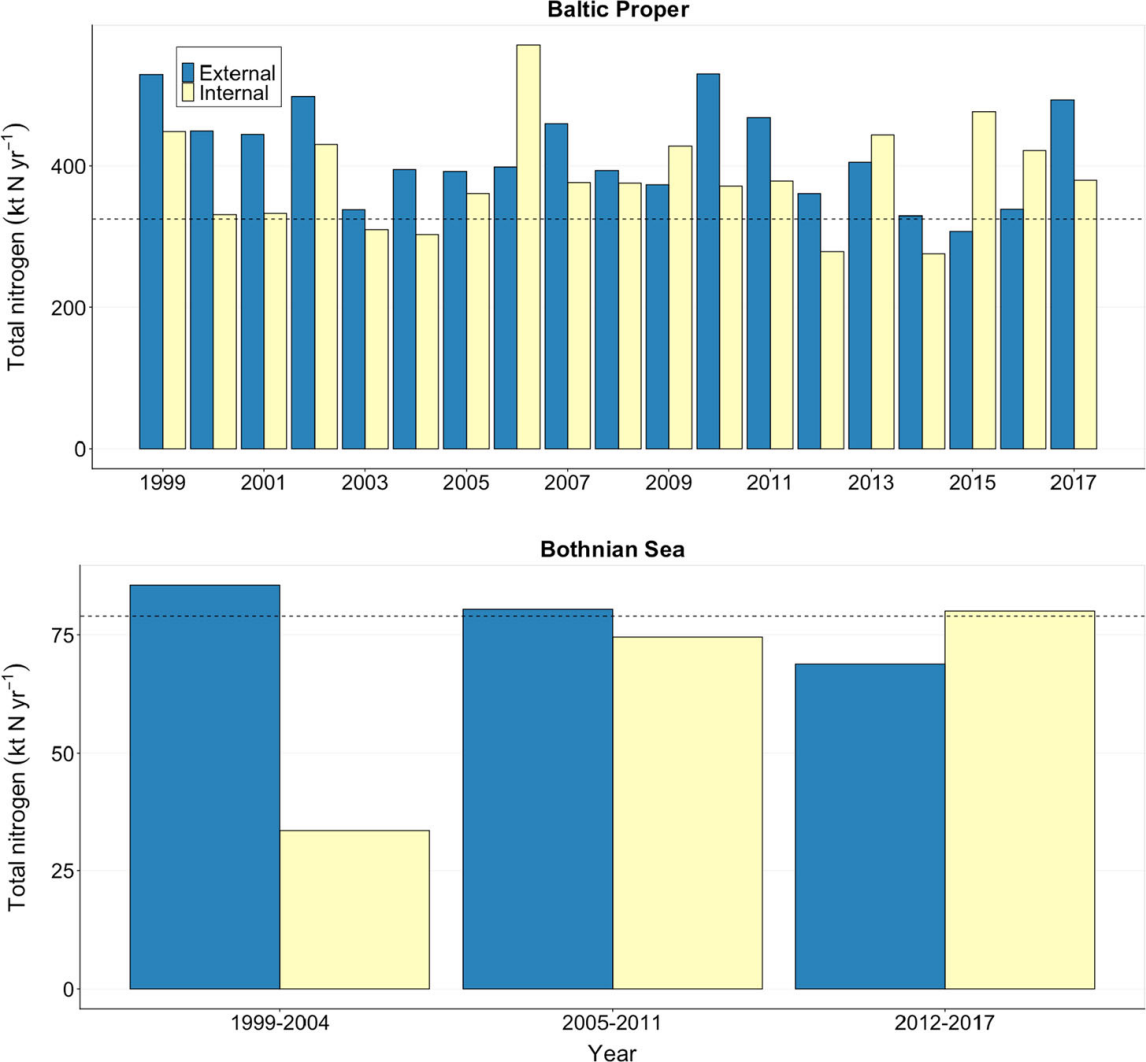
Total external input of nitrogen (from rivers, direct sources, and atmospheric deposition) in blue (for 1999–2017), and the internal input of nitrogen from estimated nitrogen fixation (kt N year^−1^) in yellow based on five stations in the Baltic Proper (upper panel), and two stations in the Bothnian Sea (lower panel). The dashed lines indicate the maximum allowable nitrogen input of 325 kt year^−1^ to the Baltic Proper and 79 kt year^−1^ to the Bothnian Sea. The external and maximal allowable nitrogen input is provided by HELCOM (2018)

The highest nitrogen fixation rates (mmol m^−2^ day^−1^) were estimated at stations BY31 and BY5, but inter-annual variability was large (Fig. 5; Eq. 1). Interestingly, station C3 in the Bothnian Sea had almost as high estimated nitrogen fixation rates (mmol m^−2^ day^−1^) as station B1 in the Baltic Proper (Fig. 5). Nitrogen fixation rates in the Bothnian Bay were lower than those of the Baltic Proper and the Bothnian Sea (Fig. 5). The average annual internal nitrogen load via nitrogen fixation was estimated to 384 ± 74 kt year^−1^ in the Baltic Proper for 1999–2017 and to an average of 399 ± 78 kt N year^−1^ during 2013–2017 (Table 3). The annual nitrogen load via nitrogen fixation in the Bothnian Sea was estimated to 34 kt year^−1^ for 1999–2004 and 80 kt year^−1^ for 2012–2017 (Fig. 6), with an overall average for 1999–2017 of 63 ± 25 kt year^−1^ (Table 3).

**Table 3:** Estimates of annual nitrogen fixation (kt year^−1^) by filamentous cyanobacteria in the Baltic Proper (211 000 km^2^) and the Bothnian Sea (79 000 km^2^) reported previously and herein. Average values between years or stations are given in brackets when available. For this study, mean values ± standard deviation (years) are included

Area	Nitrogen input	Method	Period (years)	Station(s)	References
Baltic Proper	30–260 (143)	Conceptual model	1992–1997	BY5, BCS-III, BY38, BY15, BY31, BY28, SR5	Rahm et al. (2000)
Baltic Proper	370	Budget + ^15^N_2_	1997–1998	M1, M2, K8, K5, K2, K1, J1, T1	Wasmund et al. (2001)
Baltic Proper	178–414 (296)	Budget	1994–1998	Mainly BY31	Larsson et al. (2001)
Baltic Proper	511	Budget	2005	Transect	Schneider et al. (2009)
Baltic Proper	434–792 (613)	Budget + ^15^N_2_	2001	BMP J1/BY15	Wasmund et al. (2005)
Baltic Proper	310	Budget	2002	Transect	Rolff et al. (2007)
Baltic Proper	337–820 (514)	Total nitrogen and budget	1998–2000	BY31	Gustafsson et al. (2013)
Baltic Proper	363–428 (396)	Total nitrogen and budget	2011	BY29, BY31	Svedén et al. (2016)
Baltic Proper	370 ± 64 399 ± 78 384 ± 74	Biovolume and empirical rates	1999–2003 2013–2017 1999–2017	B1, BY31, BY15, BY5, BY2	This study
Bothnian Sea	63 ± 25	Biovolume and empirical rates	1999–2017	B3 + B7, C3	This study

## Discussion

This study provides a genera-specific overview of the spatial and temporal distribution of filamentous diazotrophic cyanobacteria in the Baltic Sea during the two recent decades (1999–2017). By combining Baltic Sea monitoring data with in situ measurements of cyanobacterial activity, we present the first estimates of internal nitrogen loads (via nitrogen fixation) in the Bothnian Sea. With an average of ca. 1 mmol N m^−2^ day^−1^ in July, the estimated nitrogen fixation rate at the Bothnian Sea station C3 was similar to rates in the Baltic Proper station B1. The internal nitrogen load in the Bothnian Sea during 2012–2017 was herein estimated to 80 kt N year^−1^, which is greater than the total external input of 69 kt N year^−1^, and the HELCOM (2018) suggested allowable limit of 79 kt N year^−1^. The estimated internal nitrogen load during the recent years are twice the amount two decades ago (Fig. 6), which is in concert with a recent monitoring study demonstrating a large increase of diazotrophic filamentous cyanobacteria in the Bothnian Sea across the recent four decades (Olofsson et al. 2020). Diazotrophic cyanobacteria in the Bothnian Sea are therefore likely contributing with substantial, previously overlooked, quantities of internal nitrogen via nitrogen fixation.

In contrast to the Bothnian Sea, nitrogen fixation in the Baltic Proper has been investigated during several decades (Table 3). In the present study, the internal nitrogen load by diazotrophic cyanobacteria was estimated to 399 kt N year^−1^ during 2013–2017 for the Baltic Proper, which is greater than the total external input from rivers and atmospheric deposition of 345 kt N year^−1^. Therefore, the external and internal nitrogen inputs are equal to twice the suggested allowable load of 325 kt year^−1^ (HELCOM 2018). Although estimated nitrogen fixation rates at station C3 in Bothnian Sea and B1 in the Baltic Proper were in the same range, the overall accumulated biovolume was higher in the Baltic Proper, resulting in a higher estimated nitrogen load in the latter, not proportional to the difference in area coverage (Fig. 3). An important difference between nitrogen derived from riverine runoff and from filamentous cyanobacteria are where it enters the water. For example, along the Baltic Sea coastline, biogeochemical processes remove nitrogen from riverine input by 50–100% (Edman et al. 2018), and all of it therefore does not reach open Baltic Sea. While denitrification is a process capable of removing reactive nitrogen from a system, it is unlikely that denitrification is a significant sink of nitrogen in well-oxygenated offshore water columns (Edman et al. 2018). The diazotrophic cyanobacteria are therefore the main contributors of new nitrogen in the open Baltic Sea, along with atmospheric deposition. Their presence, activity, and future distribution is hence of importance to understand, as they are key players in this habitat.

The main advantage of our approach is the resolution down to single cyanobacterial taxa, including both empirical fixation rates and monitoring data. Genera-specific observations are of importance since the Baltic Sea diazotrophic cyanobacteria are known to perform nitrogen fixation at different rates and their community composition varies spatially (Klawonn et al. 2016). The three genera of filamentous cyanobacteria in the present study dominate the pelagic near-surface nitrogen fixation in the Baltic Sea, as neither picocyanobacteria nor *Pseudanabaena* could be confirmed to perform nitrogen fixation in Klawonn et al. (2016). However, low nitrogen fixation rates by heterotrophic bacteria have been detected in the dark, sub-surface zone (Riemann et al. 2010). Previous estimates of nitrogen fixation for the area did not account for the cyanobacterial community composition (Table 3). Yet, the mean value from the measurements by Klawonn et al. (2016; Table 2) where within the range of previously measured nitrogen fixation rates divided by cyanobacterial biovolume for station BY15 of ca. 1.3 μmol mm^−3^ day^−1^ in June 2001 (Wasmund et al. 2005) and ca. 0.74 μmol mm^−3^ day^−1^ in August 1997 (Wasmund et al. 2001). Svedén et al. (2015) reported rates of ca. 0.45 μmol N mm^−3^ day^−1^ by *Aphanizomenon* sp. already in early June in 2010. In addition, single-cell measurements for *Aphanizomenon* sp. from station B1 in August 2008 were on average ca. 0.50 μmol N mm^−3^ day^−1^ (Ploug et al. 2010) and ca. 0.51 μmol N mm^−3^ day^−1^ for *N. spumigena* in August 2009 (Ploug et al. 2011), validating our use of average genera-specific activity for estimating nitrogen fixation in the Baltic Proper. However, due to the variation in empirical rates between locations, the present approach may not be used to determine exact nitrogen fixation rates at a particular station. Since the activity is related to many factors, e.g., phosphate availability, mixing, and light, there is a need for in situ measurements in the Bothnian Sea to validate our predictions.

Another advantage of the present study is the temporal and spatial distribution of filamentous cyanobacteria across two decades. Most previous estimates are based on fewer years and/or stations (Table 3). Both Svedén et al. (2016) and Larsson et al. (2001) used nitrogen budget calculations, resulting in similar estimates for nitrogen loads as herein. Schneider et al. (2009) included early spring (as in the present study) in the budget calculations, resulting in slightly higher values as compared to other previous studies, while the conceptual model of Rahm et al. (2000) resulted in much lower values. As our estimates of nitrogen fixation within the Baltic Proper are consistent with the findings of other studies, it here extend beyond previous approaches by including the Bothnian Sea. Heterocystous cyanobacteria in the Baltic Sea are optimized for low salinities and moderate temperatures (Staal et al. 2003; Stal 2009) which limits their spread through the Danish straits, but may govern their distribution throughout the northern sub-basins of the Baltic Sea. Long-term monitoring data for the Bothnian Sea recently demonstrated a large increase of filamentous cyanobacteria in the central parts during the recent decades (Olofsson et al. 2020). The increased abundance of *Aphanizomenon* sp. may be explained by decreased salinities during the same time frame. The nitrogen fixation estimates for the Bothnian Sea herein were based on two stations in its northern regions, while diazotrophic cyanobacteria have mainly been reported on satellite images from the central and southern regions (Kahru and Elmgren 2014). As cyanobacteria blooms comprise mainly *Aphanizomenon* in the Bothnian Sea, they are supposedly largely overlooked on satellite images due to their sub-surface distribution (Hajdu et al. 2007). Complementary monitoring across the entire Bothnian Sea basin is therefore needed to predict future trends of internal nitrogen load estimates.

The availability of phosphate is known to affect the growth of cyanobacteria (Moisander et al. 2007; Olofsson et al. 2016). An increased phosphate availability has been demonstrated for both the Baltic Proper and the Bothnian Sea during recent decades (Suikkanen et al. 2007, 2013; Jaanus et al. 2011; Kahru and Elmgren 2014; Andersson et al. 2015; Kuosa et al. 2017; Wesslander et al. 2018). Rolff and Elfwing (2015) report that, in addition to elevated input of phosphate rich waters from the Baltic Proper, nitrogen availability has decreased in the Bothnian Sea during the last 20 years, which in combination may enhance blooms of diazotrophic cyanobacteria. However, a turnover time of down to hours of phosphate when at low concentrations (Nausch et al. 2018) complicates determination of the actual availability, and may partly explain the high biovolume of filamentous cyanobacteria but generally low phosphate concentrations in the Baltic Sea during summer. In addition, phosphate is not the only phosphorus source available to filamentous cyanobacteria, where a recent study demonstrates that *Aphanizomenon* sp. possibly acquire only about 15% of their phosphorus from phosphate, and the remaining from organic sources (Schoffelen et al. 2018). Phosphate availability will therefore not necessarily determine the abundance and activity of *Aphanizomenon* sp. in the Bothnian sea, where it is the dominating taxa. The nitrogen fixation rates in the Bothnian Sea need to be quantified in situ to clarify how the current nutrient situation affects these rates.

The distribution of filamentous cyanobacteria in the Baltic Sea is known to be spatially highly heterogeneous, both vertically and horizontally (Kahru et al. 1994; Wasmund et al. 2001; Rolff et al. 2007). The variation of cyanobacteria biovolume between monitoring samples (up to 200 times during summer) was larger than the empirical single-cell nitrogen fixation rates of 8 times between the two stations (Klawonn et al. 2016). The large sample variation is likely due to patchiness in the blooms and large short-term temporal variations, especially in the southern Baltic Sea where the surface accumulating *N. spumigena* dominates (Rolff et al. 2007). Rolff et al. (2007) suggested a minimum of 10 samples per estimate for this region, and therefore, we used mean values across 5 or 2 stations per basin (for Baltic Proper and Bothnian Sea, respectively), and across 6–7 years for the latter due to low sampling frequency. In addition, tube sampling provides mean biovolume over the sampling depth, which here includes most of the diazotrophic filamentous cyanobacteria (Hajdu et al. 2007). The initial conversion factor of 2 for stations sampled at 0–20 m instead of 0–10 m is valid for *N. spumigena* which is mostly found in the upper water column, while *Aphanizomenon* sp. has the deepest vertical distribution and can locally be overestimated (Walve and Larsson 2007). Nitrogen fixation rates in the Baltic Sea decrease with depth, where rates at the surface are about double those at 10 m. We use the mean activity from 0–7 to 0–12 m in situ measurements to integrate through the water column (Klawonn et al. 2016). The large pool of biovolume data in the present study (up to 77 samples for a given station and month; Table 1) further reduces variability, and the Central Limit Theorem states that, when sample size is large, sample means will approach the population mean and be normally distributed. The mean empirical single-cell rates used herein were applied on more than 2000 single field samples during almost two decades.

The total external nitrogen input (rivers and atmospheric deposition) to the Baltic Sea (Bothnian Sea, Baltic Proper, Gulf of Finland, and Gulf of Riga) was estimated to 898 kt year^−1^ in 1999 and 655 kt year^−1^ in 2016, with the suggested maximum allowable input of 658 kt year^−1^ (HELCOM 2018). The internal nitrogen load to the entire Baltic Sea is difficult to estimate, as the present study does not include observations throughout all sub-basins. Satellite images of the Gulf of Finland and Gulf of Riga indicate large surface formations in the summer (Kahru and Elmgren 2014). Monitoring data indicate even higher cyanobacterial biomass in these regions compared to the Baltic Proper stations (Wasmund et al. 2017). Applying the estimated areal nitrogen fixation rates from the Baltic Proper to the area of Gulf of Finland suggests nitrogen fixation in the Gulf is 90 kt N year^−1^. In this case, the total internal nitrogen load to the Baltic Sea equals 582 kt year^−1^, which is 0.4% of the global nitrogen fixation according to Landolfi et al. (2018) and Wang et al. (2019), but up to 1% according to Luo et al. (2012). As the Baltic Sea only covers 0.1% of the global ocean area, this is an aquatic system with one of the highest impacts by nitrogen fixation. The estimated nitrogen fixation rates during summer in the Baltic Sea of 1–2 mmol N m^−2^ day^−1^ are globally among the highest (Luo et al. 2012), being higher than those in the South Pacific of 0.6 mmol N m^−2^ day^−1^, North Atlantic of 0.2 mmol N m^−2^ day^−1^, and North Pacific of 0.1 mmol N m^−2^ day^−1^ (Landolfi et al. 2018). The rates are also well above average oceanic measurements of 0.09 mmol N m^−2^ day^−1^ and average coastal rates of 0.6 mmol N m^−2^ day^−1^ (Tang et al. 2019). Precautionary, nitrogen fixation estimates should be of increasing interest in Baltic Sea management, using basin- and taxa-specific approaches due to variable trends in cyanobacterial abundance along with changes in environmental parameters.

## Conclusion

The Baltic Sea offers a suitable case study for ecosystem management due to its long history of anthropogenic stressors and monitoring data collection (Reusch et al. 2018). If the Baltic Sea Action Plan is accomplished, both nitrogen fixation and primary production are predicted to decrease within the next 50–100 years (Saraiva et al. 2018). This plan is a good example of how management and future legislation of nutrient loads in the region could be handled, including considerations for internal nitrogen loading. While internal loads of nitrogen fixation in the Baltic Sea have been investigated for at least two decades, in situ measurements are still lacking for the Bothnian Sea. According to our estimates, both the Baltic Proper and the Bothnian Sea have external and internal nitrogen loads that combined are twice the allowable limits suggested by HELCOM, highlighting the need to include the previously neglected Bothnian Sea in future predictions. For this region, extended monitoring and empirical measurements are pivotal for accurately monitor the contribution by the filamentous cyanobacteria, as they are increasing in abundance and spreading further north (Olofsson et al. 2020). We suggest that extended monitoring in the Bothnian Sea should include additional stations in central parts of the basin (where it is now lacking), and also samplings covering the whole year. The approach used herein can be applied to future monitoring data of diazotrophic cyanobacteria and empirical species-specific nitrogen fixation rates for estimates of internal nitrogen loads, and it can also be used when tuning biogeochemical models applied for the Baltic Sea. If internal nitrogen loads continue to increase, it may counteract the positive effects of decreased external nutrient loading, allowing eutrophication to continue.
